# CarSPred: A Computational Tool for Predicting Carbonylation Sites of Human Proteins

**DOI:** 10.1371/journal.pone.0111478

**Published:** 2014-10-27

**Authors:** Hongqiang Lv, Jiuqiang Han, Jun Liu, Jiguang Zheng, Ruiling Liu, Dexing Zhong

**Affiliations:** School of Electronic and Information Engineering, Xi’an Jiaotong University, Xi’an, China; Swiss Institute of Bioinformatics, Switzerland

## Abstract

Protein carbonylation is one of the most pervasive oxidative stress-induced post-translational modifications (PTMs), which plays a significant role in the etiology and progression of several human diseases. It has been regarded as a biomarker of oxidative stress due to its relatively early formation and stability compared with other oxidative PTMs. Only a subset of proteins is prone to carbonylation and most carbonyl groups are formed from lysine (K), arginine (R), threonine (T) and proline (P) residues. Recent advancements in analysis of the PTM by mass spectrometry provided new insights into the mechanisms of protein carbonylation, such as protein susceptibility and exact modification sites. However, the experimental approaches to identifying carbonylation sites are costly, time-consuming and capable of processing a limited number of proteins, and there is no bioinformatics method or tool devoted to predicting carbonylation sites of human proteins so far. In the paper, a computational method is proposed to identify carbonylation sites of human proteins. The method extracted four kinds of features and combined the minimum Redundancy Maximum Relevance (mRMR) feature selection criterion with weighted support vector machine (WSVM) to achieve total accuracies of 85.72%, 85.95%, 83.92% and 85.72% for K, R, T and P carbonylation site predictions respectively using 10-fold cross-validation. The final optimal feature sets were analysed, the position-specific composition and hydrophobicity environment of flanking residues of modification sites were discussed. In addition, a software tool named CarSPred has been developed to facilitate the application of the method. Datasets and the software involved in the paper are available at https://sourceforge.net/projects/hqlstudio/files/CarSPred-1.0/.

## Introduction

Oxidative stress is the direct result of imbalance in the production and degradation of reactive oxygen species (ROS) and reactive nitrogen species (RNS). It arises while oxidative intermediates are exceeding detoxification ability of cells [1], [2]. Oxidative modification of cellular macromolecules, such as nucleic acids, proteins, lipids and carbohydrates, may be concomitant and all seriously deleterious. Proteins are possibly the most immediate vehicles for inflicting oxidative damage on cells, because they are often catalysts rather than stoichiometric mediators [3]. Post-translational modification (PTM) is a chemical modification of proteins, which occurs naturally and plays pivotal roles in the regulation of protein function [4]. Oxidative stress can induce various kinds of PTMs such as hydroxylation, nitration, sulfhydrylation, carbonylation and glutathionylation [5]. Among these PTMs, protein carbonylation has been widely studied and regarded as a biomarker of oxidative stress due to its relatively early formation and stability compared with other oxidative stress-induced protein modifications. Protein carbonyl researches focus currently on the development and optimization of high-throughput LC-MS-based methods to identify carbonylated proteins, modified residues and carbonylation types [6], [7], [8]. The total level of carbonylation increases with aging, obesity and external oxidative stress [9], [10]. Human diseases associated with protein carbonylation include Alzheimer's disease, chronic lung disease, chronic renal failure, inflammatory bowel disease, rheumatoid arthritis, diabetes, sepsis and so on [3], [11].

Only a subset of proteins is prone to carbonylation and most carbonyl groups are formed from lysine (K), arginine (R), threonine (T) and proline (P) residues [12]. The site-specific oxidative damage of proteins is now regarded as a major cause of metabolic dysfunction during pathogenesis [13]. Identification of the susceptible amino acid residues could provide deeper insights into the mechanisms of protein carbonylation [14]. Therefore, the identification and especially the mapping of protein carbonylation sites are crucial [2]. Mass spectrometry and liquid chromatography have been used to analyze protein susceptibility of the oxidative PTM and exact carbonylation sites recently [2], [15]. More carbonylation sites are found in RKPT-enriched regions and carbonylation sites have a strong tendency to cluster [12], [16], [17]. However, the experimental approaches are costly, time-consuming and capable of processing a limited number of proteins, and there is no bioinformatics method or tool devoted to predicting carbonylation sites of human proteins so far, the only relevant existing tool named CSPD [12] is just applicable to the Escherichia coli proteome. A new method and software tool for predicting carbonylation sites of human proteins seems useful and necessary.

In this paper, a computational method is proposed to identify carbonylation sites of human proteins based on amino acid sequences only. The human carbonylation datasets verified by experiments were collected from the literature. Four kinds of features, including position-specific propensity of amino acid and k-spaced amino acid pair, increment of k-mer diversity, k nearest neighbor (KNN) scores as well as physicochemical and biochemical properties, were extracted from sample sequences. The minimum Redundancy Maximum Relevance (mRMR) feature evaluation criterion [18] and incremental feature selection (IFS) were used to evaluate the importance of candidate features and determine the dimension of the final optimal feature sets. Then weighted support vector machine (WSVM) [19] was employed to solve the classification problem of unbalanced training samples. The final optimal feature sets were analysed, the position-specific composition and hydrophobicity environment of flanking residues of modification sites were discussed. In addition, the software tool CarSPred for win32 environment has been developed to facilitate the application of the method.

## Materials and Methods

### Datasets

Datasets involve carbonylated protein sequences and K, R, T and P carbonylation sites of human and other mammals. Because there is no relevant public database available for protein carbonylation records so far, carbonylation datasets can only be obtained by looking up experimental data in the literature. A total of 230 carbonylated protein sequences as well as 331 K, 131 R, 128 T and 129 P carbonylation sites of human were extracted from eight sources in proteomic studies. A total of 20 carbonylated protein sequences as well as 22 K, 13 R, 6 T and 15 P carbonylation sites of other mammals, such as mouse, rabbit and bovine, were also collected from four sources. For details, please refer to Table S1 and Data S1.

### Sample preparation

Protein sequences with any confused carbonylation sites were excluded (Table S2). Then datasets of human carbonylation in which all the four types of carbonylation sites have been involved were used for training sample preparation. Due to the close homology between human proteins and other mammal proteins, the testing samples were prepared from the remaining datasets of human and carbonylation data of other mammals.

#### Positive and negative sample sequences

The carbonylation sites were used as candidate positive samples. A given residue has to meet three criteria to be selected as a candidate negative sample. Firstly, a candidate negative sample must have the same residue type as known carbonylation sites and could not have been reported as a positive one. Secondly, it has to be within a protein that contained known carbonylation sites. Finally, a candidate negative sample has to be extracted from a dataset in which the same type of carbonylation site has been involved.

The ±n (n = 5 to 13) flanking residues of these candidate sites were extracted to prepare sample sequences. The window size was preset to be from 5 to 13 on the basis of the following points. Firstly, more carbonylation sites are found in RKPT-enriched regions. Around the RKPT-enriched region, a specific environment region is rich in various residues including iron-binding sites and hydrophobic amino acids. [12], [16]. The RKPT-enriched region was proposed to set to 4 residues long and the window size of environment region can be up to 29 residues in Maisonneuve’s study [12]. Rao and Moller suggested that the effective window size of RKPT-enriched region should be 7 residues [16]. Secondly, the motif of residues around carbonylation sites has been analyzed and all the four types of motifs were 21 residues long eventually [20]. Finally, the position-specific statistical differences between positive and negative sample sequences of human carbonylation involved in the study were computed using a web-based analysis application named Two Sample Logo (TSL) [21]. It is obvious that the RKPT-enriched degree in ±13 flanking residue fragments of carbonylation sites is different (see position-specific composition analysis section for details). Therefore, taking into account the strong clustering of carbonylation sites [12], [16], the window size n ranged from 5 to 13 in the paper. The CD-HIT program [22] was used with a 30% cut-off threshold to remove repetitive and excessively similar sample sequences. Then the central residue was excluded because it is always the same in both positive and negative sample sequences.

#### Sample imbalance correction

The numbers of positive and negative sample sequences were highly imbalanced, which easily resulted in inflated evaluation of the method. Therefore, we randomly chose negative sample sequences for about 6 times to match the number of positive ones. Eventually, four groups of residue-type independent training sample sets were prepared including 266 K, 119 R, 116 T and 114 P positive training sample sequences as well as 1,802 K, 754 R, 702 T and 716 P negative training sample sequences. In the same way, 34 K, 17 R, 5 T and 12 P positive testing sample sequences as well as 147 K, 93 R, 30 T and 76 P negative ones were also prepared. Datasets above are summarized in Table 1. Training and testing sample sequences are available in Data S2.

**Table 1:** Carbonylation datasets involved in this paper.

Group	Dataset	No. of carbonylated proteins	No. of carbonylation sites			
			K	R	T	P
Training samples	Original sequences	227^a^	307	126	128	129
	Positive samples	223^b^	266	119	116	114
	Negative samples	223	1,802	754	702	716
Testing samples	Original sequences	23^c^	46	18	6	15
	Positive samples	23	34	17	5	12
	Negative samples	23	147	93	30	76

a Human carbonylated proteins from proteomic studies in which all the four types of carbonylation sites were involved.

b Four proteins have been excluded. For details, please refer to Table S2.

c The remaining carbonylated proteins of human and carbonylated proteins of other mammals.

### Feature extraction

Four kinds of features were extracted from the sample sequences. In the feature extraction approach, 21 types of amino acids were considered including 20 native and one dummy amino acid X.

#### Position-specific propensity of amino acid and k-spaced amino acid pair

The position-specific amino acid propensity (PSAAP) and k-spaced amino acid pair have been successfully used in various applications and some new promising approaches of PTM site prediction [23], [24]. In the paper, the k-spaced amino acid pair is introduced into the standard PSAAP and a new feature encoding scheme called position-specific propensity of amino acid and k-spaced amino acid pair (PSPAKSAP) is proposed. In the PSPAKSAP, the feature vector of a query sample sequence is constructed by looking up the corresponding parameters in a position-specific propensity matrix. The matrix is given by:
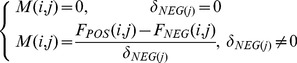
(1)where 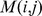 is a 21×2n and 441×2n×(2n-1)/2 matrix for amino acid and k-spaced amino acid pair respectively, in which 21 and 441 are the numbers of amino acid element types for amino acids and k-spaced amino acid pairs, and 2n is the residue length of sample sequences. 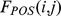 and 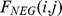 denote the absolute frequency of the 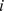 amino acid element appearing at 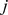 position in the positive and negative training sample sets separately. 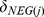 is the standard deviation of 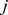 column of the absolute frequency matrix 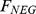. It is defined as:
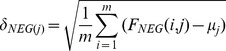
(2)where m is the total number of rows of 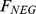, 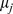 is the average value of 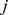 column of 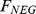.

In the section, a 2n-dimensional feature vector and a 2n×(2n–1)/2-dimensional feature vector corresponding to amino acids and k-spaced amino acid pairs were extracted separately from each sample sequence. Therefore, the dimension of PSPAKSAP-based feature vector is 2n+2n×(2n–1)/2. The PSPAKSAP vector reflects position-specific propensity of amino acid and k-spaced amino acid pair.

#### Increment of k-mer diversity

Based on the theory of the measure of diversity, the increment of diversity is a measure of the total uncertainty in a system, by which the similarity level of two datasets can be quantitatively described. The increment of k-mer diversity is the increment of diversity of k-mer residue fragments in a sample sequence.

The standard diversity measure for diversity source 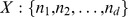 is given by [25]:
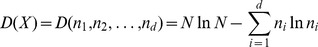
(3)where 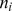 is the absolute frequency of the 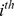 state, 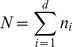.

For two diversity sources 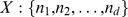 and 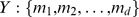, the increment of diversity is defined as:
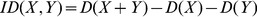
(4)where 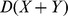 is the measure of diversity of the mixed source 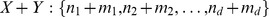. The more similar are the two sources, the smaller score is the 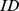.

In the paper, the difference in k-mer diversity increment of a query sample sequence with positive and negative training samples is given by:
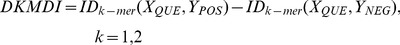
(5)where 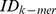 denotes the increment of k-mer diversity, 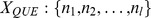
is a diversity vector based on query sample sequence, 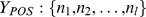 and 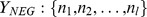 are diversity vectors based on the positive and negative training samples. 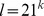, that is to say there are a total of 21 and 441 states when k is set to 1 and 2 respectively.

In the section, the increment of k-mer (k = 1, 2) diversity was used to generate a 2-dimensional feature vector for each sample sequence. It is obvious that the dimension of the vector has nothing to do with the residue length of sample sequences, because the increment of k-mer diversity indicates composition information of k-mer fragments in a sequence and has no relationship with k-mer fragment position.

#### KNN scores

The KNN algorithm has been widely applied to text categorization due to its simple, valid and non-parameter advantage. Recently, the KNN has also been introduced into the prediction of PTM sites [26]. To get the KNN score, firstly, calculate average distances from a query sample to both positive and negative training sample sequences; secondly, sort the neighbours by the distances; finally, calculate the KNN scores which is the percentage of positive neighbours in a number of its KNNs. In this section, the average distance was based on the BLOSUM62 substitution matrix [27] and the dummy amino acid X would be ignored. Chosen k (k = 10n+1, n = 1, 2, ···, 5) as different values of the number of neighbours to obtain multiple features. Therefore, a 5-dimentioanl feature vector could be extracted for each sample sequence. The KNN describes the similarity of the flanking sequence around a possible carbonylation site to training sample sequences.

#### Physicochemical and biochemical properties

AAIndex is useful in many approaches of PTM site prediction. However, the sheer number of properties could potentially cause both computational tractability and overfitting problems in a classification approach [28]. Recently, a set of high-quality indices (HQI) were identified to represent electric properties, hydrophobicity, alpha and turn propensities, physicochemical properties, residue propensity, composition, beta propensity and intrinsic propensities of amino acids using a sophisticated method called consensus fuzzy clustering [29]. In the section, the normalized HQI8 was considered for each residue of a given sample sequence to generate a 2n×8-dimentional feature vector. The parameter value corresponding to residue X was set to 0. This vector reflects physicochemical and biochemical properties of the residue fragments around a query carbonylation site.

### mRMR and IFS feature selection

The mRMR feature selection criterion is devoted to evaluating the importance of candidate features, which ranks the features according to their relevance to the target concerned and the redundancy among the features themselves. The ranked feature with a smaller index indicates that it has a better trade-off between the maximum relevance and minimum redundancy [30]. In this study, four kinds of features were extracted from each sample sequence. The total number of these candidate features is 2n+2n×(2n–1)/2+2+5+2n×8. For example, there are a total of 566 candidate features when the window size parameter n = 13. These features were ranked according to mRMR feature selection criterion. The IFS curve was used to determine the dimension of the final optimal feature sets.

### WSVM classifier

The support vector machine (SVM) is a supervised machine learning algorithm based on the statistical learning theory [31]. The basic thought of SVM is to map the original data into a high dimensional feature space through a nonlinear mapping function and then construct a hyperplane as a discriminative surface between positive and negative samples. In this paper, the WSVM was employed to solve the classification problem of unbalanced samples, which is available at http://www.csie.ntu.edu.tw/~cjlin/libsvm/.

### Performance assessment

The jack-knife test and n-fold cross-validation are usually used to assess the performance of a method. Jack-knife test is a leave-one-out cross-validation approach, which is a special case of n-fold cross-validation. Breiman and Spector found that 5-fold and 10-fold cross-validation work better than jack-knife test [32]. In the paper, 10-fold cross-validation was used to illuminate the performance of our method. Five standard metrics, including specificity, sensitivity, total accuracy, Matthew’s correlation coefficient (MCC) and the area under the Receiver Operating Characteristic curve (AUC) [33], were adopted to quantitatively evaluate the predictive capability and reliability of the method.

True positive (TP) and false negative (FN) are the number of positive data that are predicted to be positive and negative respectively. Analogously, true negative (TN) and false positive (FP) are used to denote the number of negative data that are predicted to be negative and positive respectively. The specificity (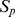), sensitivity (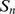), total accuracy (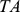) and 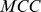
are defined as the following:
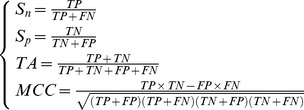
(6)


## Results and Discussion

### Quality of datasets

Data quality has an impact on evaluation of the performance of a method. In the paper, the following steps have been taken to try to improve quality of datasets. Firstly, data sources. Carbonylation sites verified by the experiments were extracted from sources as much as possible (Table S1). Secondly, exclusion of suspicious data. Protein sequences with any confused carbonylation sites were excluded (Table S2). Thirdly, preparation of negative samples. There are three criteria for negative sample preparation. The rationale is that residues are not known to be carbonylated are more likely to be true non-carbonylation sites in a proteomic study, in which the same type of carbonylation site has been involved. Finally, construction of non-redundant datasets. Repetitive and excessively similar sample sequences have been removed. Carbonylation sites can be verified by experiments. However, assignment of negative cases can only be tentative, as new experimental evidence may reveal them to be carbonylated under a different condition. Although not all these samples are absolutely true, it is reasonable to believe that a large majority of them are.

### Final optimal feature sets

**Figure 1:**
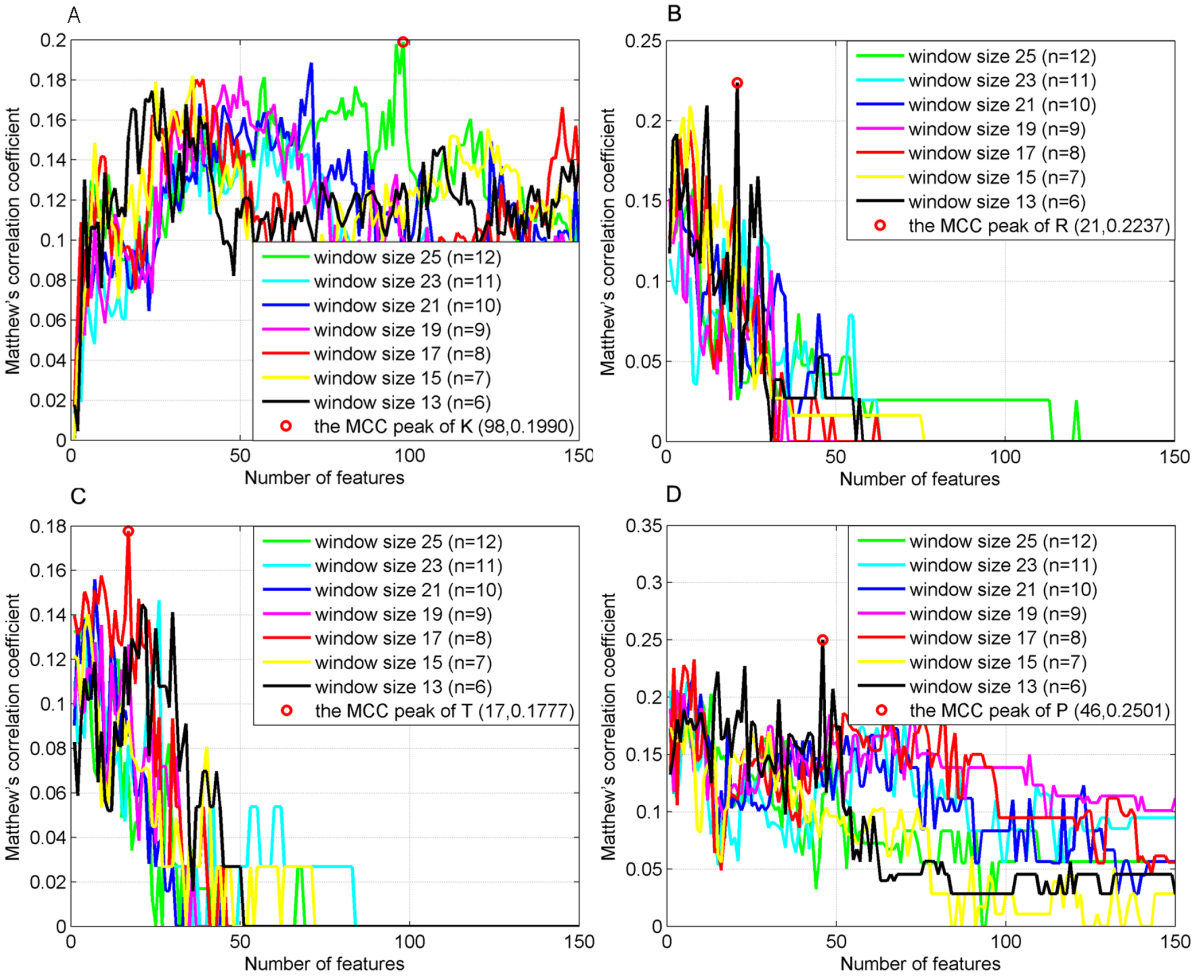
Change of average MCC values versus the number of mRMR features and different window sizes using 10-fold cross-validation (n = 6 to 12 only). (A) K carbonylation site prediction, (B) R carbonylation site prediction, (C) T carbonylation site prediction and (D) P carbonylation site prediction.

To determine the final optimal feature sets of the method, the IFS curves of average MCC value versus the number of mRMR features and different window sizes were plotted using 10-fold cross-validation based on the training datasets (Fig. 1). It is obvious that the MCC values corresponding to K, R, T and P carbonylation site predictions reach the peaks when n = 12, n = 6, n = 8 and n = 6 as well as the top 98, 21, 17 and 46 features were selected separately. Therefore, the four final optimal feature sets were eventually chosen to devote to K, R, T and P carbonylation site identification respectively. Considering that too many curves easily make a figure unclear, there are only seven (n = 6 to 12) necessary IFS curves were shown in Fig. 1.

### Performance of the method

**Figure 2:**
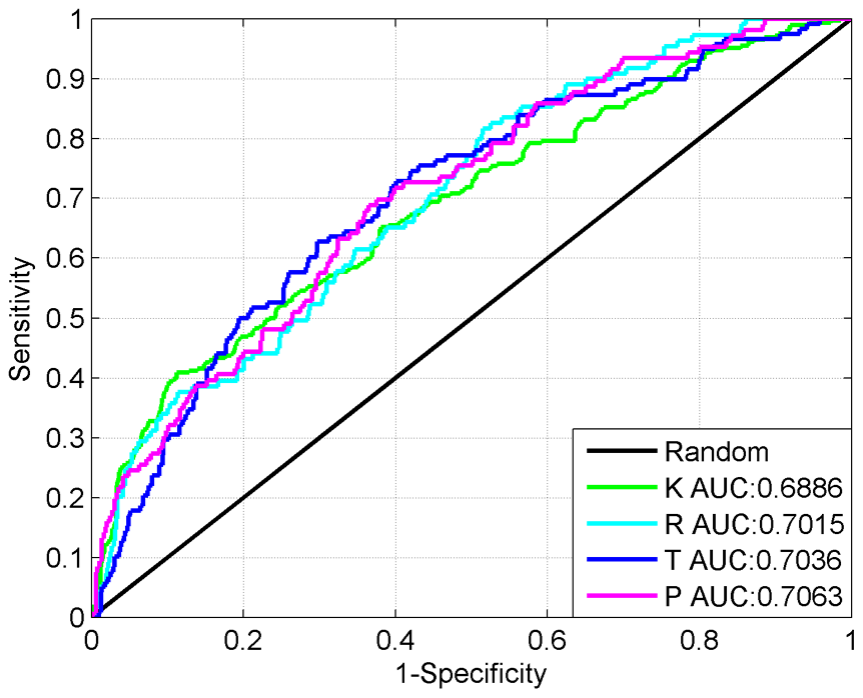
ROC curves of the method corresponding to K, R, T and P carbonylation site predictions using 10-fold cross-validation.

In the paper, the proposed WSVM classifier was trained and tested using 10-fold cross-validation based on the training datasets. The probability results of the ten iterations were spliced into one to serve the total accuracy computation and ROC analysis. The proposed method has total accuracies of 85.72%, 85.95%, 83.92% and 85.72% for K, R, T and P carbonylation site predictions respectively. ROC curves of the four types of carbonylation sites were plotted in Fig. 2, the AUC values corresponding to K, R, T and P carbonylation site identification were 0.6886, 0.7015, 0.7036 and 0.7063 separately.

Following the evaluation using 10-fold cross-validation, the method was further evaluated based on the independent testing samples. The total accuracies corresponding to K, R, T and P carbonylation site predictions were 81.22%, 85.45%, 88.57% and 86.36%. And the AUC were 0.6704, 0.5345, 0.6800 and 0.7873 respectively. It can be seen that, due to the close homology between human proteins and other mammal proteins, the method can also be used to predict carbonylation sites of other mammal proteins to a certain extent.

Overall, the predictive power of the method is still weak. In addition to the method itself, it may be due to the following reasons. Firstly, assignment of negative carbonylation sites can only be tentative, some carbonylatable residues, which may be revealed to be carbonylated under a different condition in subsequent proteomic studies, are assumed to be negative samples. Secondly, to some degree, the limitation of training sample size might affect the validity of absolute frequency-dependent features involved in the paper.

### Feature analysis

**Figure 3:**
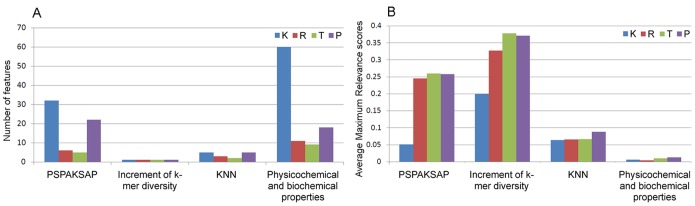
The distribution of the four kinds of features in the K, R, T, and P optimal feature sets. (A) The number of each kind of features in the optimal feature sets. (B) The average Maximum Relevance scores of the four kinds of features in the optimal feature sets.

The distribution of the number of each kind of features in the K, R, T and P optimal feature sets was investigated and shown in Fig. 3A. In the 98 optimal features devoted to the prediction of K carbonylation sites, 32 were from the PSPAKSAP, 1 from increment of k-mer diversity, 5 from KNN scores, and 60 from physicochemical and biochemical properties. Likewise, all the four kinds of features have made contributions to the predictions of R, T and P carbonylation sites. However, the total dimensions of the four kinds of features are very different, only the number of each kind of features in the optimal feature sets is not enough to rely on. Therefore, the average Maximum Relevance scores of the four kinds of features in the optimal feature sets were computed (Fig. 3B). Two types of scores can be generated by mRMR program, one is mRMR score and the other is the Maximum Relevance score. The final optimal feature sets consist of four kinds of features, these features could be divided into four groups. The average Maximum Relevance score of each kind of features is an average value of Maximum Relevance scores of features in the corresponding group. Considering the number and average Maximum Relevance score, it was inferred that the PSPAKSAP plays a more important role in the carbonylation site prediction.

### Position-specific composition analysis

The position-specific sequence characteristics are important conserved features in the mRMR feature list. Therefore, the web-based analysis application TSL [21] was used to analyse the position-specific composition of amino acids surrounding carbonylation and non-carbonylation sites. TSL was running with default parameter options, two sample *t*-test was chosen and the P-value threshold was assigned to 0.05. The statistical significance was calculated for each residue in the flanking sequences of modification sites and graphically represented. Statistically significant residues were plotted using the size of the residue symbol that is proportional to the difference of position-specific composition of amino acids between positive and negative samples. Graphical residues were separated in two groups, enriched and depleted in the positive samples.

**Figure 4:**
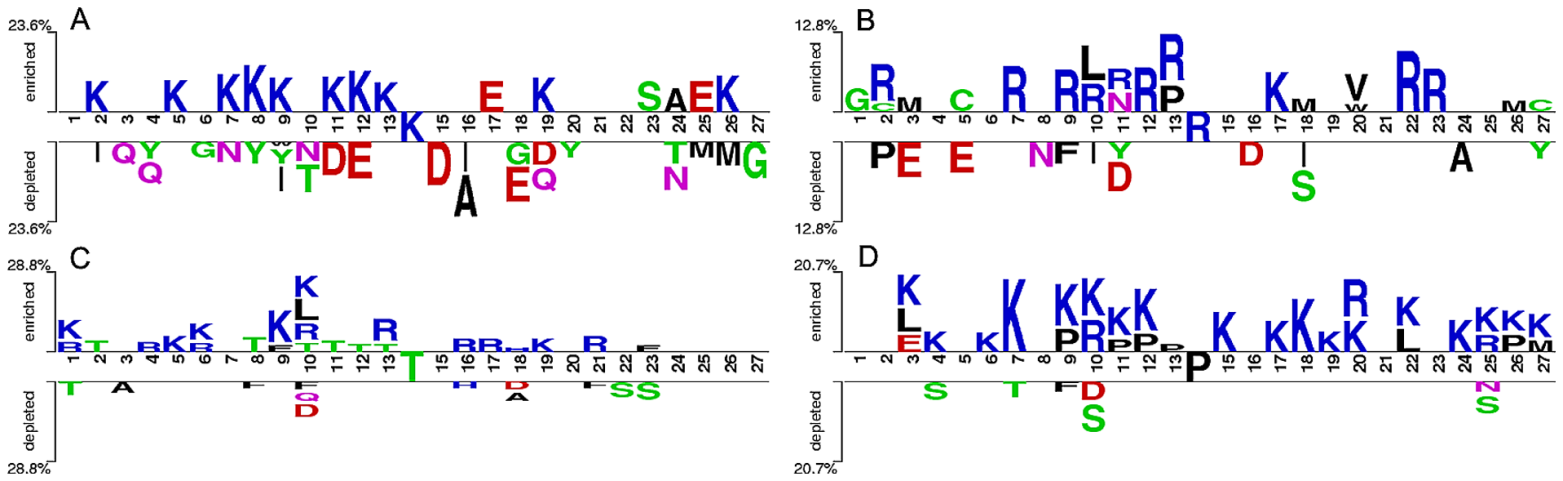
Two-Sample-logos of the position-specific composition of residues surrounding carbonylation and non-carbonylation sites. It shows position-specific residues enriched and depleted in positive samples of (A) K carbonylation site prediction, (B) R carbonylation site prediction, (C) T carbonylation site prediction and (D) P carbonylation site prediction, respectively.

The position-specific statistical differences between positive and negative sample sequences of human carbonylation sites were given in Fig. 4. It is obvious that these differences vary according to the modification residue type, but the K, R, T and P carbonylation sites are all prone to be in RKPT-enriched regions, which is consistent with Maisonneuve and Rao’s studies. [12], [16]. It is noteworthy that the work by Maisonneuve was designed for the prokaryotic Escherichia coli and more species were involved later in Rao’s study. So this is likely to be an important and general rule for carbonylation sites. Furthermore, it can be seen from Fig. 4 that the enrichment degree of K residue in the upstream of K carbonylation site is high than that in the downstream, and likewise the R, T and P carbonylation sites. Therefore, for the residue with the same type as the carbonylation site,the enrichment degree in the upstream is significantly high than that in the downstream.

### Hydrophobicity environment analysis

**Figure 5:**
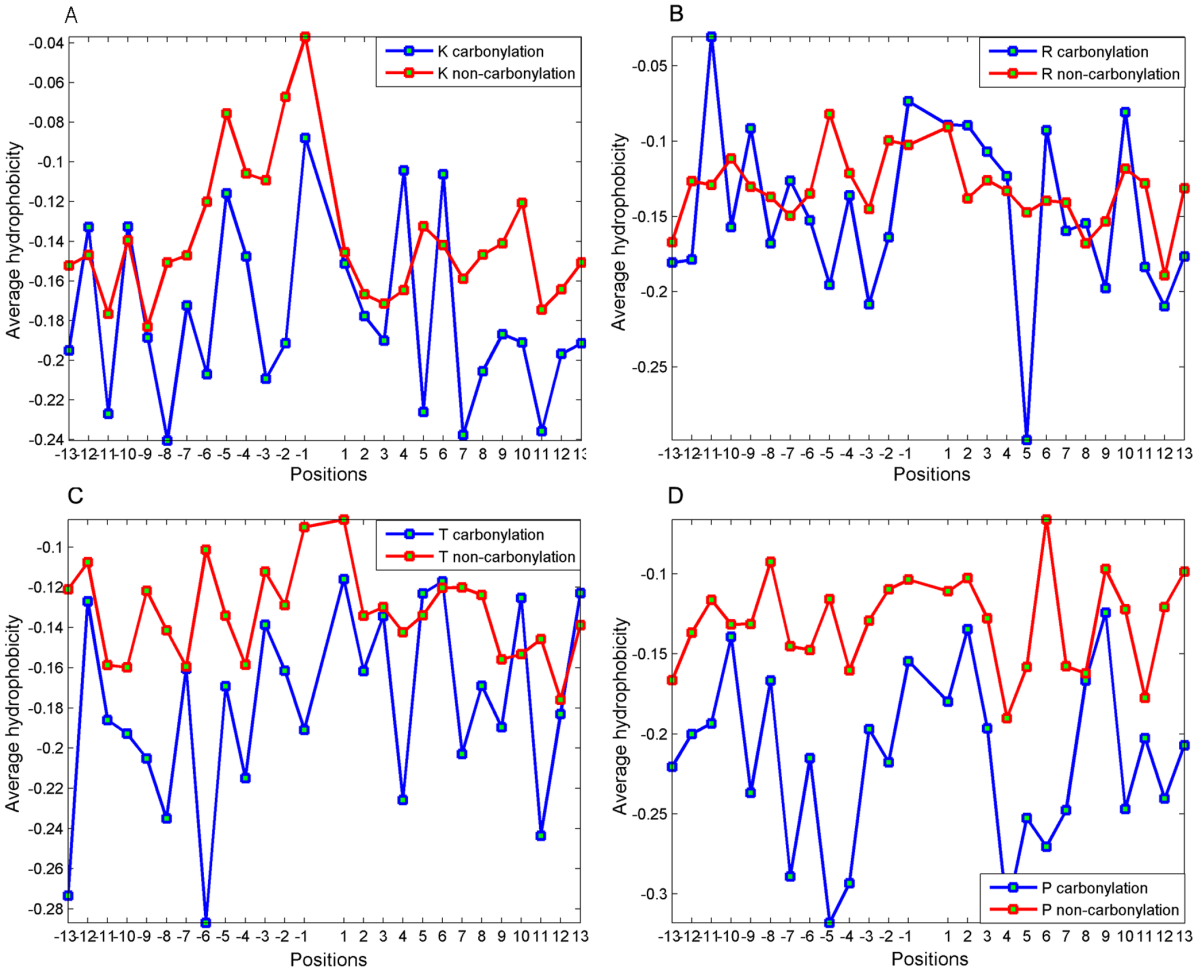
The average hydrophobicity at each position (excluding the carbonylation site itself) around carbonylation and non-carbonylation sites. (A) K carbonylation site prediction, (B) R carbonylation site prediction, (C) T carbonylation site prediction and (D) P carbonylation site prediction.

The hydrophobicity environment of carbonylation sites has been discussed in the previous work [12], [16]. It may play an important role in protein carbonylation. Therefore, average hydrophobicity at each position around carbonylation and non-carbonylation sites was computed using the normalized hydrophobicity index of HQI (Fig. 5). It can been seen that hydrophobicity environment of carbonylation sites is not significantly different from non-carbonylation sites, which is in line with Rao’s study [16]. However, the hydrophobicity difference at certain upstream positions (−6∼−2) is remarkable for all the K, R, T and P carbonylation sites. This investigation proves that the informative HQI can contribute to distinguishing carbonylation and non-carbonylation sites. The position-specific difference of hydrophobicity still needs further verification due to the sample size limitation.

### CarSPred software

In order to facilitate the application of this method, software named CarSPred 1.0 for win32 environment has been developed. This tool consists of four modules which are devoted to K, R, T and P carbonylation site predictions of query protein sequences separately. All of them receive sequences or file in FASTA format as input. For output, list and file are optional, the annotations will clearly indicate the precise locations and probabilities of putative carbonylation sites. For details, please refer to the manual coming with the software.

## Supporting Information

Table S1 Carbonylation sites of human and other mammal proteins collected from the literature.
(DOC)

Table S2 Carbonylation sites and their corresponding proteins identified in proteomic studies but excluded in this paper.
(DOC)

Data S1 Carbonylated proteins involved in the paper.
(XLSX)

Data S2 Training and testing sample sequences corresponding to K, R, T and P carbonylation site predictions.
(XLSX)
