# Supplementary material for: CarSPred: A Computational Tool for Predicting Carbonylation Sites of Human Proteins
Source: PLoS One. 2014 Oct 27;9(10):e111478. doi: 10.1371/journal.pone.0111478 (PMC4210226; doi:10.1371/journal.pone.0111478)
Supplement: Table S1 — Carbonylation sites of human and other mammal proteins collected from the literature. (DOC) [file pone.0111478.s001.doc]

**Table S1.** Carbonylation sites of human and other mammal proteins collected from the literature.

| Group | Species | Reference | Year | No. of  proteins | No. of carbonylation sites | | | | Use |
| --- | --- | --- | --- | --- | --- | --- | --- | --- | --- |
|  |  |  |  |  | K | R | T | P |  |
| Human | Human |  | 2014 | 211 | 276 | 119 | 119 | 116 | Train |
|  | Human |  | 2011 | 7 | 7 | 3 | 2 | 3 | Train |
|  | Human |  | 2010 | 7 | 9 | 1 | 4 | 1 | Train |
|  | Human |  | 2008 | 1 | 6 | 0 | 0 | 0 | Test |
|  | Human |  | 2006 | 1 | 7 | 0 | 0 | 0 | Test |
|  | Human |  | 2006 | 1 | 12 | 2 | 1 | 6 | Train |
|  | Human |  | 2006 | 1 | 3 | 1 | 2 | 3 | Train |
|  | Human |  | 2006 | 1 | 11 | 5 | 0 | 0 | Test |
| **Total** | | | | **230** | **331** | **131** | **128** | **129** |  |
| Other mammals | Mouse |  | 2011 | 13 | 12 | 3 | 1 | 2 | Test |
|  | Mouse |  | 2005 | 5 | 1 | 4 | 0 | 1 | Test |
|  | Rabbit |  | 2010 | 1 | 2 | 2 | 0 | 2 | Test |
|  | Bovine |  | 2009 | 1 | 7 | 4 | 5 | 10 | Test |
| **Total** | | | | **20** | **22** | **13** | **6** | **15** |  |

**References**

1. Bollineni RC, Hoffmann R, Fedorova M (2014) Proteome-wide profiling of carbonylated proteins and carbonylation sites in HeLa cells under mild oxidative stress conditions. Free Radic Biol Med 68: 186-195.

2. Madian AG, Diaz-Maldonado N, Gao Q, Regnier FE (2011) Oxidative stress induced carbonylation in human plasma. J Proteomics 74: 2395-2416.

3. Madian AG, Regnier FE (2010) Profiling carbonylated proteins in human plasma. J Proteome Res 9: 1330-1343.

4. Ishii T, Ito S, Kumazawa S, Sakurai T, Yamaguchi S, et al. (2008) Site-specific modification of positively-charged surfaces on human serum albumin by malondialdehyde. Biochem Biophys Res Commun 371: 28-32.

5. Temple A, Yen TY, Gronert S (2006) Identification of specific protein carbonylation sites in model oxidations of human serum albumin. J Am Soc Mass Spectrom 17: 1172-1180.

6. Mirzaei H, Regnier F (2006) Identification and quantification of protein carbonylation using light and heavy isotope labeled Girard's P reagent. J Chromatogr A 1134: 122-133.

7. Mirzaei H, Regnier F (2006) Enrichment of carbonylated peptides using Girard P reagent and strong cation exchange chromatography. Anal Chem 78: 770-778.

8. Lee S, Young NL, Whetstone PA, Cheal SM, Benner WH, et al. (2006) Method to site-specifically identify and quantitate carbonyl end products of protein oxidation using oxidation-dependent element coded affinity tags (O-ECAT) and nanoliquid chromatography Fourier transform mass spectrometry. J Proteome Res 5: 539-547.

9. Madian AG, Myracle AD, Diaz-Maldonado N, Rochelle NS, Janle EM, et al. (2011) Determining the effects of antioxidants on oxidative stress induced carbonylation of proteins. Anal Chem 83: 9328-9336.

10. Mirzaei H, Regnier F (2005) Affinity chromatographic selection of carbonylated proteins followed by identification of oxidation sites using tandem mass spectrometry. Anal Chem 77: 2386-2392.

11. Chavez JD, Bisson WH, Maier CS (2010) A targeted mass spectrometry-based approach for the identification and characterization of proteins containing alpha-aminoadipic and gamma-glutamic semialdehyde residues. Anal Bioanal Chem 398: 2905-2914.

12. Maisonneuve E, Ducret A, Khoueiry P, Lignon S, Longhi S, et al. (2009) Rules governing selective protein carbonylation. PLoS One 4: e7269.
