# Supplementary material for: CarSPred: A Computational Tool for Predicting Carbonylation Sites of Human Proteins
Source: PLoS One. 2014 Oct 27;9(10):e111478. doi: 10.1371/journal.pone.0111478 (PMC4210226; doi:10.1371/journal.pone.0111478)
Supplement: Table S2 — Carbonylation sites and their corresponding proteins identified in proteomic studies but excluded in this paper. (DOC) [file pone.0111478.s002.doc]

**Table S2.** Carbonylation sites and their corresponding proteins identified in proteomic studies but excluded in this paper.

| Group | Reference | Year | Protein  accession | No. of carbonylation sites | | | | Reasons for excluding |
| --- | --- | --- | --- | --- | --- | --- | --- | --- |
|  |  |  |  | K | R | T | P |  |
| Human |  | 2014 | sp|Q9BPX7 | 1 | 0 | 1 | 0 | Reason 1^a^ |
|  |  | 2014 | sp\|Q9UHC1 | 2 | 1 | 0 | 1 | Reason 1 |
|  |  | 2011 | sp\|P02751 | 1 | 0 | 0 | 0 | Reason 2^b^ |
|  |  | 2011 | sp\|P23083 | 0 | 1 | 0 | 1 | Reason 2 |

^a^The central residue type of sample sequences is arginine (R), but was found in the lysine (K) carbonylation site subgroup. The corresponding sample sequences are ‘TKLRKMAQAWRLFGKAECDTR’ and ‘IKRAESLSRSRKGGIEGGAKL’.

^b^Residue type of annotated carbonylation site is inconsistent with the corresponding residue of designated protein sequence in NCBI.

**References**

1. Bollineni RC, Hoffmann R, Fedorova M (2014) Proteome-wide profiling of carbonylated proteins and carbonylation sites in HeLa cells under mild oxidative stress conditions. Free Radic Biol Med 68: 186-195.

2. Madian AG, Diaz-Maldonado N, Gao Q, Regnier FE (2011) Oxidative stress induced carbonylation in human plasma. J Proteomics 74: 2395-2416.
